# Placing and preserving priorities: projects, productivity, progress and people

**DOI:** 10.1155/S1463924698000170

**Published:** 1998

**Authors:** John Babiak

**Affiliations:** Wyeth-Ayerst Research Robotics and Automation CN 8000 Princeton New Jersey 08543 USA

## Abstract

High throughput screening (HTS) involves using automated equipment to test a large number of samples against a defined molecular target to identify a reasonable number of active molecules in a timely fashion. Major factors which can influence priorities for the limited resources of the HTS group are projects, productivity, progress and people. The challenge to the HTS group is to provide excellent and timely screening services, but still devote efforts to new technologies and personnel development. This article explains why these factors are so important.


Journal of Automatic Chemistry, Vol. 20, No. 4 (July–August 1998) pp. 117–120

### Placing and preserving priorities: projects, productivity, progress and people*

**John Babiak**

*Wyeth-Ayerst Research, Robotics and Automation, CN 8000, Princeton, New Jersey 08543, USA*

*High throughput screening (HTS) involves using automated equipment to test a large number of samples against a defined molecular target to identify a reasonable number of active molecules in a timely fashion. Major factors which can influence priorities for the limited resources of the HTS group are projects, productivity, progress and people. The challenge to the HTS group is to provide excellent and timely screening services, but still devote efforts to new technologies and personnel development. This article explains why these factors are so important.*

### Introduction

Automated high throughput screening (HTS) in the pharmaceutical industry has become a valuable resource to search for proprietary chemical structures which interact with novel biomolecular targets such as enzymes, cellular receptors or ion channels. Under some circumstances these chemicals may serve as leads which can be optimized into marketable drugs. To maximize success it is necessary to use HTS resources in a manner which complements and facilitates the changing priorities of drug discovery research. The enormous medical benefits associated with bringing a novel drug to market provide an incentive to complete all steps in the drug discovery process, including HTS, as quickly and efficiently as possible.

Numerous institutions and individuals involved in the discovery and development of a new drug possess unique, changeable and sometimes conflicting priorities. Some of these participants include the FDA, the marketplace, research departments within the pharmaceutical company, individual scientists and HTS team members. Since it is never possible to do everything—in HTS or any other aspect of real life—priorities are needed to make the best choices. Four major factors which can influence priorities are:

- Projects.
- Productivity.
- Progress.
- People.

Long-term success depends upon creating a balance among all these factors. The purpose of this paper is to highlight the importance of each in providing an excellent high throughput screening service within a large pharmaceutical company.

* Presented at the International Symposium on Laboratory Automation and Robotics, Boston, MA, 19–22 October, 1997.

### Projects—choosing the best molecular target

The human genome consists of tens of thousands of gene products—proteins such as enzymes, receptors, ion channels and growth factors—which are important for maintaining good health, if not life itself. Many of these gene products are likely to play a role in disease and, therefore, have the potential to be a molecular target for pharmacological intervention. Before HTS resources can be applied to discover chemicals which can interact with any of these specific molecular targets, the decision must be made as to which gene products represent the best targets to pursue.

The process of initiating a project around a particular target (or targets) is difficult to generalize. Some drug discovery research groups may be organized around a therapeutic area, such as cardiovascular or inflammatory diseases, while others might focus on classes of molecular-based mechanisms such as 7-transmembrane domain receptors or signal transduction cascades. A wide range of factors, from proprietary scientific expertise through existing market franchises, must be evaluated to determine which general classes of targets to examine. Even if a general area is accepted as important for pharmacological intervention, there is no single approach to identify the best molecular target. For example, the many enzymes involved in cholesterol synthesis were well known and studied for decades before the first successful drug which inhibited only one of the enzymes was discovered.

Once a specific molecular target has been selected, a great deal of hard work is necessary to determine how best to apply HTS resources to find leads which modulate the activity associated with this target. Research might indicate that a particular gene—for example, a cell-surface receptor—is found in greater abundance in certain diseases. A high throughput screen might be created which looks for molecules which decrease the transcription of the gene for this receptor. Or, a screen might be created which seeks molecules which prevent the binding of the natural ligand to this receptor. Or, a screen might be created which inhibits the intracellular messages that occur subsequent to activation of the cell surface receptor. In other words, even if a single interesting molecular target can be identified, there are several potential screening approaches available for testing and these options need to be prioritized.

Setting priorities should take advantage of the three perspectives required to create a successful HTS, namely:

- Pharmacology and physiology.
- Biophysics and biochemistry.
- Automation and robotics.

The three perspectives on HTS do not agree on all matters, but each is essential to success.

0142-0453/98 $12.00 © 1998 Taylor & Francis Ltd

The pharmacology and physiology views must come from the scientists in the therapeutic area who are familiar with the pathologic role of the chosen molecular target. It is useful to know the conditions under which the target protein functions. For example, knowing the concentrations of natural ligands, substrates and cofactors in both the healthy and diseased states is important because they reflect the conditions under which the future drug must work. This type of information may suggest, for example, that inhibition of ligand binding is likely to be a more effective therapy that reduction in transcription.

Biophysics and biochemistry contribute to determining the feasibility of assembling a valid assay which can be developed into a high throughput screen. It is essential that the interactions be examined in detail to understand the effects of time, temperature and reagent concentrations on kinetic parameters, such as initial rates of enzymes or equilibrium binding constants. These metrics can be used both to maintain consistently high quality of different batches of key reagents as well as to select assay conditions which mimic the true pathologic situation and minimize interassay variability.

The automation and robotics perspective takes into account the available HTS resources to produce a screen with maximum signal to noise which can be performed with maximum throughput. This may require elimination of tedious manual steps, or temperature changes, which might produce artifacts such as condensation or uneven heating rates of samples with microtitre plates.

The three perspectives on HTS (pharmacology and physiology; biophysics and biochemistry, automation and robotics) may recommend different assay designs, but each must make compromises to create a single screen which can be ultimately successful.

Prioritizing a high throughput screen based upon the target (the project) makes sense in light of the scientific strengths and marketing goals of the pharmaceutical company. When the best target is identified, it is still necessary to know enough about the circumstances of the disease state and the biophysical properties of the target protein to understand how to design a viable assay. The nature of the assay must impact the priority of the project, however, because some screens are easier to perform or are best suited to the available HTS people and hardware.

### Productivity—research by numbers

High throughput screening groups are generally evaluated on the basis of productivity numbers. The most common measures are:

- *Screens*–the number run per year or how many are performed simultaneously.
- *Time lines*—time to develop a screen, finish it or deliver results.
- *Throughput*—the number of samples tested per week.
- *Samples*—the total number of entities (compounds or extracts) tested.
- *Hit rate*—the number of active samples found compared to expected rate.

Although all of these are measures of productivity, they are not always indicative of accomplishment or scientific contribution.

It is easy to identify the factors which have contributed to the perception that HTS achievements can be reduced to productivity metrics. First, the sight of robotic arms moving in a laboratory gives a strong visual impression of productivity. In addition, unrealistic throughput claims are often made by hardware vendors, screening service providers and HTS team members to justify the large financial investment required by large scale HTS. Most obvious of all, the genuine success of HTS groups themselves, sometimes demonstrating several-fold increases in data output within a few years, has served to elevate expectations for equivalent increases in the future.

The emphasis on numbers perpetuates two false assumptions: that all assays can be run with equal efficiency on all robots, and that the only job of the HTS group is to run assays. Only people who work in a laboratory within a pharmaceutical company are aware of the great variety of processes that could be referred to as a screen and that some screens are more amenable to automation than others. Biological components could include cells, enzymes, receptors, ion channels, transcription factors or microorganisms, each of which has its own requirements for handling and stability. At the biochemical level, a screen may consist of an enzymatic reaction, binding of a receptor to a ligand or a cellular activity (including death). Performance of the screen may require pipetting of different samples of different volumes, mixing, washing, centrifugation, filtration and incubation with regulation of temperature, humidity and atmospheric gases. The physical endpoint measured might be radioactivity, spectrophotometry, luminescence, fluorescence, polarized fluorescence or time resolved fluorescence. It is unlikely that a single robotic system, even an entire laboratory, could be prepared to meet all the possible contingencies the next high-priority screen may demand. It is likely, however, that an HTS group could perform many of the permutations of screen designs described above. This diversity of capability, however, will come at the expense of throughput. The true measure of success of the HTS group, therefore, is not merely the quantity of data generated, but also the diversity of automation applications implemented which meet the needs of as many individual scientist customers as possible.

The second incorrect assumption is that all the robotic high throughput screening group does is run screens. In reality, members of the robotic screening group are likely to some extent, to repair broken equipment, give tours, program and integrate robots and other automated workstations, talk to scientist customers, analyse data, order reagents, make presentations at project meetings, evaluate new equipment and software, explore new technologies for future screens, develop the automated method for the next screen and always be on call if something goes wrong.

If numbers are the primary benchmark, all activities other than running screens must be eliminated to avoid the appearance of low productivity. To create a more balanced view of productivity, additional metrics should be considered including:

- Diversity of screens performed.
- Low personnel turnover.
- Publications and presentations by HTS staff.
- Positive scientist customer feedback.
- Teaching non-HTS scientists to apply automation.
- Active new technology development projects.
- Performing screens previously considered not feasible.

Although it is necessary to generate more data against more targets to increase the likelihood of finding more chemical leads for development, many other factors contribute to create a successful and productive HTS group.

### Progress—doing the job better

While productivity measures how much of the job is being done, progress is a measure of how productivity is being improved. For HTS, this might be seen as bringing more screens on line faster and running them with greater throughput. A more general view of progress would be any improvements in the infrastructure which supports the HTS process.

The HTS infrastructure consists of the elements needed for optimal productivity, which include:

- Sample sourcing and plate handling.
- Screen format and design
- Robotic hardware
- Data management

Each of these elements plays a critical role in efficient and effective HTS. Most importantly, a deficiency in one aspect of the infrastructure could easily eliminate the value of achievement in all other areas.

The impact of sample sourcing and plate handling becomes more critical as test sample collections continue to grow through combinatorial chemistry and sample acquisitions. Test sample plates need to be stored in an appropriate manner, typically tightly sealed and frozen, but the process of thawing and unsealing individual microtitre plates is slow and usually manual. Bar coding of each plate should be done in a way that is machine-readable for real-time sample tracking, but also people-readable for rapid recognition and sorting.

It is difficult for a small HTS group to perform efficiently the large variety of possible screen formats and physical endpoints. One option is to develop excellent biochemical and automation expertise in a subset of standard assay designs which could encompass most screens likely to be encountered. Examples might include enzyme assays with spectrophotometric or fluorescent endpoints, ELISA assays and selected radioactive formats. These core competency formats can serve as examples for future HTS customers of how to create an assay which can rapidly become a high throughput screen.

Robotic and automated hardware, in addition to being reliable, must be integrated and utilized in a way which improves productivity. In this regard, the common arrangement with a central robotic arm carrying individual microtiter plates among workstations is not optimal. It is necessary to make robots work smarter, not harder. Utilization of self-feeding devices such as stackers, elevators and conveyors can increase throughput whether used manually or robotically. In addition scheduling of both hardware and people can be modified to recover time normally devoted to non-critical steps.

Diversity is what makes data management especially difficult. Readers produce data in diverse formats with non-standard headers. Data may consist of single or multiple point determinations. Controls may be a single well or a complete titration curve, possibly on a different microtitre plate from test samples. A unified approach to data management is needed to overcome this diversity and streamline processing. Data files from all readers should be converted to a standard format with well defined headers which include sample and screen identity, intended calculation scheme, unique sequence number and time stamp. Processing the results from each microtitre plate should occur as soon as the plate is read as well as standard, automated quality assessments.

### People—how it all happens

The best people in the HTS group usually have a diverse set of skills and are very broadly trained. They must routinely adapt to new ideas and face new challenges in biochemical assay design, software, data management, hardware trouble shooting and customer service. During the early development of a novel screen they are expected to take risks to find creative, efficient solutions, but during screening they ought to be very consistent and stable. They must handle all complaints from scientist customers with speed and courtesy.

The scientist customers with which the HTS group interact are, however, also people. They often require patient education about HTS from HTS personnel. Scientist customers are also under pressure because of their own and their department's priorities.

### In conclusion—a few priorities

The fundamental challenge to the HTS group is to provide excellent and timely screening services, but still devote efforts to new technologies and personnel development. To achieve this, priorities for the HTS group might be divided into short-term and middle-term priorities.

Short-term priorities need to be focused on speed and throughput. The easiest screens should be performed quickly, even at the expense of more difficult assays. It is these simpler formats which are the basis for the core competencies. The only exceptions are, of course, the few screens assigned the highest priority by senior management. Customer interactions need to be very frequent such that all news, good or bad, is delivered now.

Middle-term priorities are focused on development of the infrastructure, the people and scientist customers. Improvements to the infrastructure will always be required to meet the expanding demand for HTS. This can only be accomplished by letting the HTS staff find creative solutions. The people in HTS always need to learn more and gain more experience. In time, the emphasis on a breadth of knowledge will be replaced by examples of excellent depth of knowledge to solve the most difficult problems. Last of all, since HTS is frequently a service function, it is essential to educate the scientist customer about efficient assay design and the opportunities afforded by appropriate uses of automation.

